# Exploring the selective gray matter profile of autism spectrum disorder through Bayes Factor Modeling

**DOI:** 10.1192/j.eurpsy.2022.1642

**Published:** 2022-09-01

**Authors:** D. Liloia, F. Cauda, L. Uddin, J. Manuello, L. Mancuso, R. Keller, T. Costa

**Affiliations:** 1 University of Turin, Psychology, Turin, Italy; 2 University of California, Psychiatry And Biobehavioral Sciences, Los Angeles, United States of America; 3 Adult Autism Centre, Asl To Unit, Torino, Italy

**Keywords:** reverse inference, cerebellum, default-mode network, structural MRI

## Abstract

**Introduction:**

Despite decades of brain MRI research demonstrating atypical neuroanatomical substrate in patients with autism spectrum disorder (ASD), it remains unclear whether and to what extent disorder-selective neuroanatomical abnormalities occur in this spectrum. This, and the fact that multiple brain disorders report a common neuroanatomical substrate, makes transference and the application of neuroimaging findings into the clinical setting an open challenge.

**Objectives:**

To investigate the selective neuroanatomical alteration profile of the ASD brain, we employed a meta-analytic, data-driven, and *reverse inference*-based approach (i.e.; Bayes fACtor mOdeliNg).

**Methods:**

Eligible voxel-based morphometry data were extracted by a standardized search on BrainMap and MEDLINE databases (849 published experiments, 131 brain disorders, 22747 clinical subjects, 16572 x-y-z coordinates). Two distinct datasets were generated: the ASD dataset, composed of ASD-related data; and the non-ASD dataset, composed of all other clinical conditions data. Starting from the two unthresholded activation likelihood estimation (ALE) maps, the calculus of the Bayes fACtor mOdeliNg was performed. This allowed us to obtain posterior probability distributions on the evidence of brain alteration specificity in ASD.

**Results:**

We revealed both cortical and cerebellar areas of neuroanatomical alteration selectivity in ASD. Eight clusters showed a selectivity value *≥* 90%, namely the bilateral precuneus, the right inferior occipital gyrus, left lobule IX, left Crus II, right Crus I, and the right lobule VIIIA (Fig. 1).

**Conclusions:**

The identification of this neuroanatomical pattern provides new insights into the complex pathophysiology of ASD, opening attractive prospects for future neuroimaging-based interventions.

**Disclosure:**

No significant relationships.

